# Implementation of a spaced-repetition approach to enhance undergraduate learning and engagement in paediatrics

**DOI:** 10.3389/fmed.2025.1601614

**Published:** 2025-07-30

**Authors:** Keta Vagha, Sonali Choudhari, Amar Taksande, Jayshree Tembhurne, Jayant Vagha, Sunita Vagha

**Affiliations:** ^1^Department of Pediatrics, Jawaharlal Nehru Medical College, Datta Meghe Institute of Higher Education and Research, Wardha, India; ^2^Department of Community Medicine, Jawaharlal Nehru Medical College, Datta Meghe Institute of Higher Education and Research, Wardha, India; ^3^Department of Pathology, Jawaharlal Nehru Medical College, Datta Meghe Institute of Higher Education and Research, Wardha, India

**Keywords:** spaced repetition, undergraduate medical education, paediatrics, knowledge retention, engagement, active learning

## Abstract

**Background:**

Knowledge retention is crucial in medical education, yet traditional study methods often fail to ensure long-term recall. Spaced repetition, a cognitive learning strategy that reinforces information at increasing intervals, has shown promise in enhancing retention and engagement. While widely used in self-directed learning, its role in structured medical education remains underexplored. This study evaluates the effectiveness of spaced repetition in improving knowledge retention and student engagement in undergraduate paediatric education, offering insights into its potential for enhancing learning outcomes.

**Objectives:**

This study aimed to evaluate the effectiveness of spaced-repetition in improving knowledge retention and student engagement in undergraduate paediatric education.

**Method:**

A quasi-experimental study was conducted over 6 months at the Department of Paediatrics, Jawaharlal Nehru Medical College, Sawangi (Meghe). Ninety final-year medical students were divided into intervention and control groups. The intervention group used digital flashcards following spaced-repetition intervals of 1, 3, 7, 14, and 28 days. The control group followed traditional study methods. Knowledge retention was assessed using pre-tests and post-tests, each containing 20 multiple-choice questions. Data were analysed using paired and independent *t*-tests. Qualitative feedback from the intervention group was thematically analysed to assess engagement and motivation.

**Results:**

Pre-test scores were similar between groups (intervention: 11.42; control: 11.58, *p* = 0.0573). Post-test scores showed significant improvement in the intervention group (16.24) compared to the control group (11.89, *p* < 0.0001). The intervention group showed a significant increase in scores (*p* < 0.0001), while no significant change was seen in the control group (*p* = 0.1138). Over 90% of students in the intervention group reported improved retention, engagement, and confidence.

**Conclusion:**

Spaced repetition significantly enhanced knowledge retention and engagement in undergraduate paediatric education. This method offers a promising approach to improve long-term learning outcomes in medical education.

## Introduction

The landscape of undergraduate medical education is evolving, yet it often remains rooted in traditional pedagogical methods. These methods typically involve intensive, concentrated study sessions followed by extended periods without reinforcement. This approach can be particularly problematic in fields such as paediatrics, where continuous learning and skill development are essential (1).

The current model of undergraduate medical education in paediatrics often fails to address the cognitive limitations of learners. Intensive study sessions may lead to initial understanding, but without ongoing reinforcement, the retention of this knowledge diminishes over time. This gap in retention can be particularly detrimental in paediatrics, where the rapid acquisition and application of knowledge are crucial. The spaced repetition method has been shown to counteract the forgetting curve, a concept first introduced by Ebbinghaus (2) in 1885, which describes how information is lost over time when there is no attempt to retain it.

Spaced-repetition is an educational technique grounded in cognitive psychology and supported by extensive research. Ebbinghaus’s (2) early work laid the foundation for understanding the benefits of spaced learning on memory retention. Modern studies have reinforced these findings. For example, research by Cepeda et al. (3) demonstrated that spaced repetition significantly improves long-term retention compared to traditional massed practice. In the realm of medical education, Kerfoot et al. (4) found that spaced education markedly enhanced the retention of clinical knowledge among medical students.

Despite the robust evidence supporting spaced repetition, its application in undergraduate medical education, particularly within paediatrics, remains limited. This gap in application presents an opportunity for targeted research. Existing literature highlights the potential benefits of spaced repetition but also underscores the need for further validation within specific medical disciplines and educational levels (5, 6).

By incorporating spaced repetition into the undergraduate paediatric curriculum, this study aims to bridge existing gaps in traditional teaching methodologies and explore its effectiveness in enhancing medical education. Given the well-documented benefits of spaced repetition in improving long-term knowledge retention and recall, its application in paediatric education holds significant potential (7).

Through a structured implementation and evaluation, we seek to assess its impact on student’s understanding of key paediatric concepts, engagement levels, and motivation to learn. Additionally, by gathering qualitative feedback, we aim to gain insights into student’s experiences and perceptions of this approach. Ultimately, this study aspires to contribute to the growing body of evidence supporting spaced repetition in medical education and provide a scalable framework that can be adapted to improve learning outcomes in paediatrics and beyond.

## Methodology

This study was conducted in the Department of Paediatrics at Jawaharlal Nehru Medical College, Maharashtra, India, over a two-month period from September 2024 to October 2024, aligning with the clinical posting schedule of final-year MBBS students. This was a quasi-experimental study designed to evaluate the impact of Spaced Repetition, using *custom-designed digital flashcards*, on knowledge retention compared to only traditional learning methods.

### Study participants

The primary inclusion criterion was all final-year Part II MBBS students posted in the Department of Paediatrics during the study period who provided informed consent. Students who did not consent or were absent for any major component of the study (pre-test, didactic lecture, intervention sessions, or post-test) were planned to exclude. Attendance records were maintained throughout the study. The study comprised 90 final year students who were asked to attend all sessions due to their relevance to their academic progression. Complete attendance was achieved throughout the entire study period.

Over the two-month study period, two batches underwent their clinical postings. The first batch included 46 students; of these, the first 23 students were allocated to the intervention group, while the subsequent 23 were assigned to the control group. The second batch consisted of 44 students, with the first 22 assigned to the intervention group and the remaining 22 to the control group.

- The *intervention group* consisted of students exposed to spaced repetition using custom-designed digital flashcards in addition to traditional learning methods.
- The *control group* comprised students who followed only traditional learning methods, such as clinic notes and textbooks.

### Sampling method

Convenience sampling was employed, as the study utilized students already scheduled for clinical postings during the study period.

### Content selection and validation

Two critical paediatric topics, Developmental Milestones and Immunization were selected based on their relevance to undergraduate medical curriculum and frequent application in clinical practice. The content was validated by a panel of three senior faculty members with over 10 years of teaching experience each in paediatrics and medical education. Validation criteria included accuracy, clarity, clinical relevance, and alignment with CBME guidelines.

The validated content was then transformed into customised digital flashcards by a research team comprising four members (one Associate Professor, one Professor, one Senior Resident, and one final-year postgraduate student) under supervision, ensuring both pedagogical and technological appropriateness.

### Study tool

The multiple-choice questions (MCQs) used in the pre-test and post-test were designed to align with the university examination pattern in terms of difficulty distribution and question types. A total of 20 MCQs were included, categorized by difficulty level into easy (12), difficult (6), and very difficult (2) questions. In terms of question type, 17 were recall-based, while 3 focused on applied or problem-solving skills. This structured approach ensured that the assessment tool was representative of actual academic requirements, effectively evaluating both foundational knowledge and the ability to apply concepts in clinical scenarios.

### Implementation of the study

1. Pre-test

At the start of the clinical posting, all participants underwent a pre-test consisting of 20 MCQs designed to assess their baseline knowledge of the two selected topics: Developmental Milestones and Immunization. The pre-test questions followed the university examination pattern for distribution, ensuring an accurate baseline for evaluating the intervention’s effectiveness.

1. Didactic lecture

Following the pre-test, all participants attended a structured didactic lecture covering both topics was delivered. These sessions covered essential concepts, clinical applications, and case-based discussions related to the two topics. It provided a uniform foundational understanding for both the intervention and control groups before introducing further learning methods.

1. Intervention

- Intervention group

Students received customised digital flashcards created by the research team. These flashcards included different sets comprising both recall-based and scenario-based questions with variable levels of difficulty. The students were guided to review these flashcards at progressively increasing intervals (1, 3, 7, 14, and 28 days) in line with spaced repetition principles. The reviews were conducted during their clinical posting hours with supervision by faculty from the research team to ensure adherence. Different sets of flashcards were used across all review sessions to reinforce learning effectively.

- Control group

Students in the control group continued with traditional learning methods, which included self-study using clinical notes, standard textbooks, and guidance from faculty during clinical postings.

1. Post-test

At the end of the clinical posting, all participants completed a 20-item post-test (same as the pre-test). The MCQs in the post-test were aligned with the university examination pattern. The post-test evaluated student’s knowledge retention, comprehension, and ability to apply concepts in clinical contexts. This structured approach ensured comparability between groups and emphasized the relevance of the MCQs to real-world academic scenarios.

1. Feedback collection

After the post-test, structured feedback was collected from the intervention group using a validated feedback questionnaire framed by the research team and reviewed by the senior faculty member. The questionnaire comprised 15 items assessed on a 5-point Likert scale to evaluate effectiveness, engagement, motivation, and satisfaction. The Likert scale options were: *Strongly Agree*, *Agree*, *Neutral*, *Disagree*, and *Strongly Disagree*. Additionally, two open-ended questions were included to gather qualitative insights.

**Figure 1:**
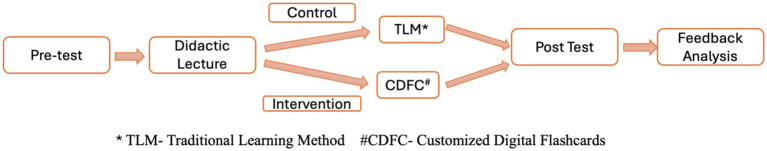
Methodology of the study.

At the end of the study, the students in the control group were given these Customised Digital Flashcards to improve their performance in the future exams. The flowchart outlining the study methodology is depicted in Figure 1.

### Faculty involvement

Multiple faculty members were involved at different stages of the study to ensure academic rigor, content validity, and methodological integrity. The multiple-choice questions used in the pre-test and post-test were designed by an Associate Professor from the research team to align with university examination standards. The customised digital flashcards were prepared by the research team in consultation with an expert panel comprising senior faculty members, including a Professor with 12 years of subject experience, who also reviewed the feedback questionnaire to ensure its content validity.

The didactic lecture for both the intervention and control groups was delivered by a senior faculty member with over 15 years of teaching experience, who was not involved in the intervention implementation or data analysis. This ensured consistency in content delivery across groups while maintaining blinding to minimise bias.

During the implementation phase, different faculty members were assigned specific roles to prevent contamination between groups. Faculty members from the research team supervised the intervention group during their spaced repetition sessions to ensure adherence to the review schedule and clarify academic doubts. In contrast, the control group was supervised by other faculty members who were not involved in the intervention to avoid inadvertent exposure to intervention materials.

This structured and clearly delineated faculty involvement ensured unbiased supervision, maintained the integrity of the intervention, and upheld methodological robustness throughout the study.

### Data analysis

Quantitative data were analysed using SPSS, with independent samples *t*-tests comparing pre-test and post-test scores between the control and intervention groups, while paired samples *t*-tests assessed within-group changes. The analysis revealed a statistically significant improvement in the intervention group. Descriptive statistics, including frequencies and percentages, were used to analyse participant feedback, highlighting positive perceptions of the spaced-repetition method. The statistical approach ensured a robust evaluation of the intervention’s impact on learning outcomes. *P* value of less than 0.05 was considered to be significant.

### Ethical considerations

The study was conducted in accordance with ethical guidelines for research involving human participants. Approval from the Institutional Ethics Committee (IEC) was obtained prior to its initiation [DMIHER(DU)/IEC/2024/332]. Written informed consent was obtained from all participants, ensuring voluntary participation and the maintenance of confidentiality.

## Results

This section presents the findings of the study, comprising both quantitative and qualitative data analysis. The quantitative analysis focuses on comparing pre-test and post-test scores to evaluate the impact of the intervention, while the qualitative analysis highlights participant feedback on the effectiveness, engagement, motivation, and overall satisfaction with the spaced-repetition method. Together, these results provide a comprehensive understanding of the intervention’s efficacy in enhancing paediatric learning outcomes.

### Quantitative data analysis

#### Participant characteristics and test results

The average pre-test score for all participants was 11.01, with the control group scoring slightly higher (11.58) than the intervention group (11.42). The *p*-value (0.0573) indicates no significant difference, confirming comparable baseline knowledge levels between the groups.

Post-test scores showed an overall improvement, with all participants averaging 14.04. However, the control group’s modest increase to 11.89 reflects limited knowledge retention with traditional methods. In contrast, the intervention group’s significant increase to 16.244 demonstrates the superior effectiveness of the spaced-repetition method. The *p*-value (<0.0001) confirms a statistically significant difference between the groups, highlighting the intervention’s impact.

Both groups started with comparable pre-test scores (*p* = 0.0573), but the intervention group showed a significantly greater improvement in post-test scores (16.244 vs. 11.89, *p* = <0.0001). This highlights the superior efficacy of the spaced-repetition method in enhancing knowledge retention and understanding of paediatric topics (see Table 1).

**Table 1:** Comparison of participant characteristics and test results.

Test results	All participants (*n* = 90)	Control (*n* = 45)	Intervention (*n* = 45)	*p*-value
Pre test	11.01	11.58	11.42	0.0573
Post test	14.04	11.89	16.244	**<0.0001**

Bold value significant <0.05.

#### Pretest and Post test results

The control group showed a minor improvement from a pre-test average of 11.58 to a post-test score of 11.89. The *p*-value (0.1138) indicates that this change is not statistically significant, suggesting traditional teaching methods had minimal impact on knowledge retention.

In the intervention group, the pre-test score of 11.42 improved dramatically to a post-test score of 16.244. The *p*-value (<0.0001) confirms this change is statistically significant, highlighting the effectiveness of the intervention, likely due to its use of spaced repetition or active learning strategies Table 2.

The comparison illustrates a stark contrast in the impact of teaching methodologies. While the control group experienced negligible gains, the intervention group’s significant improvement underscores the potential of innovative teaching strategies to enhance learning outcomes effectively.

**Table 2:** Comparison of pretest and post test results within participant groups.

Participants	Pre test	Post test	*p*-value
Control (*n* = 45)	11.58	11.89	0.1138
Intervention (*n* = 45)	11.42	16.244	**<0.0001**

Bold value significant <0.05.

### Qualitative feedback analysis

The graphical representation of the qualitative feedback analysis highlights a strong positive reception of the spaced-repetition method across all key domains. In terms of effectiveness, a majority of participants agreed that spaced repetition significantly improved retention (64.44%), made it easier to recall paediatric concepts (66.67%), reinforced understanding of key topics like developmental milestones and immunization (71.11%), and provided better long-term retention than traditional methods (66.67%). Regarding engagement and convenience, most participants found the method enjoyable (64.44%) and engaging (66.67%), with flashcards being easy to integrate into their study routine (62.22%) and the spaced intervals proving effective (62.22%).

**Figure 2:**
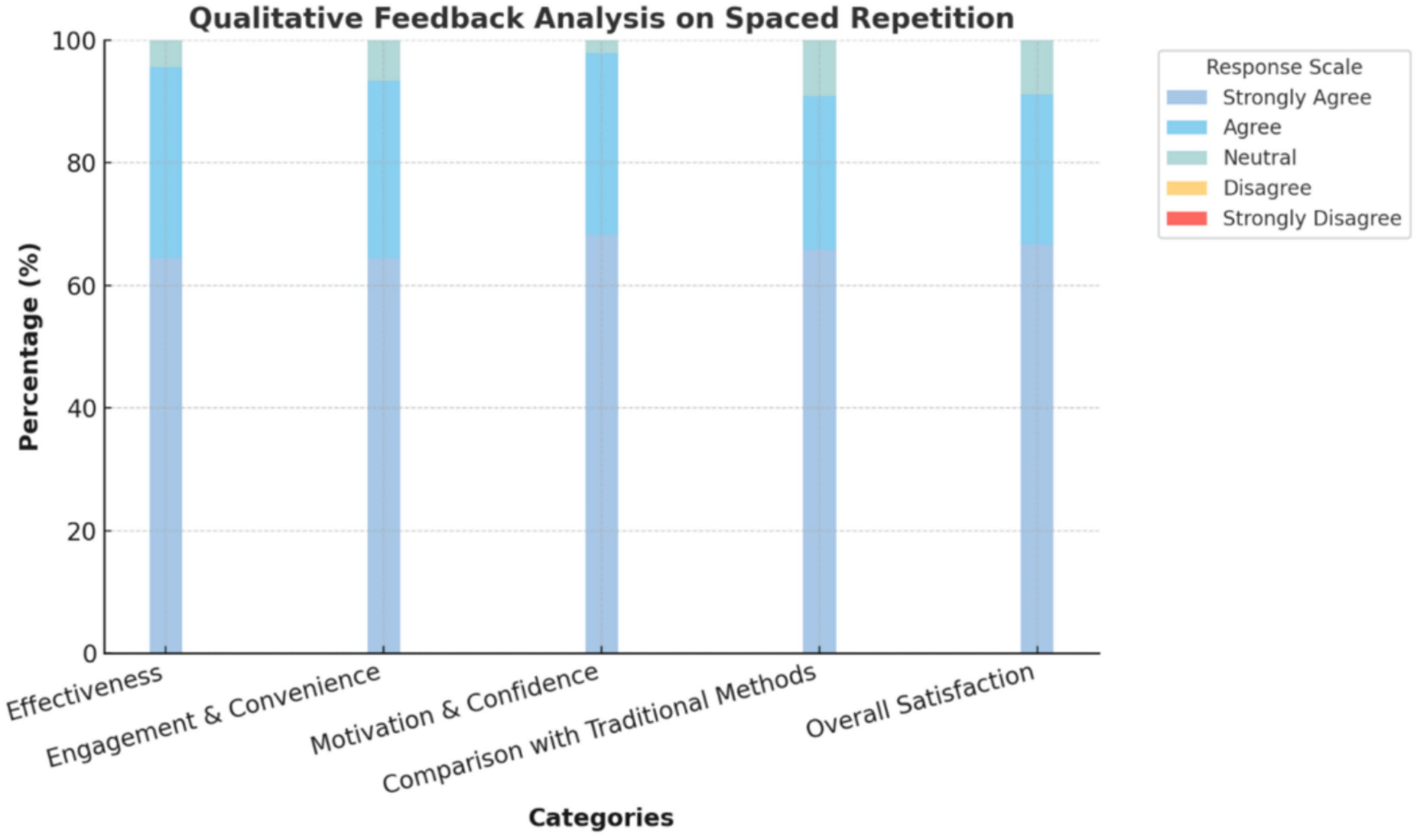
Qualitative feedback analysis on spaced repetition by the intervention group (*n* = 45).

The method also played a crucial role in motivating participants to maintain regular study sessions (62.22%), enhancing their engagement during clinical postings (64.44%), and boosting their confidence in answering questions during clinical rounds (68.18%). When compared to traditional learning methods, most respondents found spaced repetition to be more effective (65.91%), though traditional methods were still considered useful in complementing their understanding (64.44%). Overall, the majority of participants expressed satisfaction with the spaced-repetition approach (66.67%) and recommended its use in other subjects (64.44%). The minimal presence of neutral or disagreeing responses underscores the method’s strong acceptance and effectiveness in paediatric education Figure 2.

## Discussion

This study examines the effectiveness of spaced-repetition techniques in enhancing learning outcomes, knowledge retention, and student engagement among undergraduate medical students in paediatrics. The results demonstrate that spaced repetition significantly surpasses traditional learning methods, offering a viable solution to the limitations of conventional pedagogical strategies.

The findings strongly support the role of spaced repetition in improving knowledge retention. The intervention group, which utilized custom-designed digital flashcards with systematically increasing review intervals, achieved significantly higher post-test scores (16.24 ± 2.37) than the control group (11.89 ± 2.94), with a statistically significant *p*-value of <0.0001. These results highlight the effectiveness of spaced repetition in counteracting the forgetting curve by reinforcing information at optimal intervals, a principle grounded in Ebbinghaus’s memory theory.

These results align with previous research. Gilbert et al. (8) found that students using spaced-repetition software like Anki scored 6.2 to 10.7% higher on standardized exams compared to those using traditional study methods. Similarly, Durrani et al. (9) reported a statistically significant improvement in test performance among medical students utilizing flashcards, with a mean increase of 2.93 points (*p* < 0.01). Traditional learning methods, which often rely on massed learning or cramming, fail to support long-term retention, a limitation that is particularly concerning in paediatrics, where sustained recall of developmental milestones and dosages is essential.

Traditional methods, including lectures and textbooks, establish foundational knowledge but do not effectively mitigate forgetting. Spaced repetition complements these approaches by systematically reintroducing information, ensuring long-term accessibility. Additionally, it enhances student engagement and motivation. In qualitative feedback, 64.44% of students in the intervention group reported that the method was enjoyable and engaging, promoting a consistent study routine. This observation is consistent with Gilbert et al. (8), who noted that students using spaced-repetition tools experienced increased confidence and preparedness for exams. The interactive nature of digital flashcards and scheduled reinforcement likely contributed to this heightened engagement.

Moreover, 62.22% of students found spaced-repetition flashcards easy to integrate into their study schedules. Given the demanding nature of medical education, this accessibility makes spaced repetition a practical tool for reinforcing learning. The ease of use and flexibility of digital flashcard platforms, such as Anki, further emphasize their utility, as confirmed by other studies (9, 10).

While traditional teaching remains essential, spaced repetition offers a valuable adjunct. The forgetting curve limits the long-term efficacy of traditional approaches, whereas spaced repetition systematically revisits critical information, reinforcing knowledge retention. In this study, 64.44% of students reported that spaced repetition complemented their conventional learning, helping to retain key concepts and prevent knowledge gaps. This hybrid approach aligns with Tabibian et al. (10), who demonstrated that computationally optimized spaced-repetition schedules enhance learning efficiency. However, McConnery et al. (11) identified challenges such as time constraints and inconsistent adherence that may limit its independent use.

A structured integration of spaced repetition into medical curricula can enhance traditional methods by ensuring sustained retention and application. Future research should explore its long-term impact on clinical performance and investigate how AI-driven adaptive learning can optimize spaced-repetition schedules. Addressing logistical barriers such as time management will be key to ensuring its widespread adoption in medical education.

### Limitations

- A long-term follow-up to assess sustained knowledge retention beyond the immediate post-test period could not be conducted due to the limited duration of the project. As the study was aligned with the clinical posting schedule, it was not feasible to evaluate whether the improved retention observed was maintained over subsequent months.
- Although the intervention showed promising short-term results, a follow-up assessment at six months would have provided a more comprehensive understanding of its long-term effectiveness in consolidating knowledge and supporting clinical application. Such assessments are recommended for future studies to validate enduring benefits.
- Additionally, since students in the intervention group were aware of their participation, there is a potential for performance bias, as their motivation to engage with the learning material may have been influenced by the awareness of being part of a study. This is a common limitation in educational intervention research where blinding is often not feasible.

## Conclusion

In conclusion, this study highlights the superior effectiveness of spaced repetition compared to traditional learning methods alone in improving knowledge retention, student engagement, and motivation in undergraduate paediatric education. The integration of spaced repetition with traditional methods offers a promising approach to addressing the cognitive limitations of learners and ensuring better retention of critical concepts in paediatrics. The hybrid approach leveraging both techniques emerges as a key recommendation for enhancing learning outcomes in medical education. Future research should continue to explore the broader applicability and long-term effects of spaced-repetition methods across various medical disciplines.

## Data Availability

The datasets presented in this study can be found in online repositories. The names of the repository/repositories and accession number(s) can be found in the article/supplementary material.
